# Association between dietary inflammatory index and energy-adjusted dietary inflammatory index and constipation in US adults

**DOI:** 10.1186/s12876-024-03307-7

**Published:** 2024-07-25

**Authors:** Xuelian Zhao, Xiaoyu Wang, Longfang Quan

**Affiliations:** 1 Graduated School, Tianjin University of Traditional Chinese Medicine, Tianjin, 301617 China; 2 Graduated School, Shandong University of Traditional Chinese Medicine, Shandong, 250355 China; 3 Department of Anorectal Research, China Academy of Chinese Medical Sciences Xiyuan Hospital, Beijing City, 100091 People’s Republic of China

**Keywords:** Stool consistency, DII, E-DII, Constipation, NHANES, Bristol stool form scale

## Abstract

**Background:**

Diet and inflammation are associated with constipation. Dietary inflammation index (DII) and energy-dietary inflammation index (E-DII) have not been evaluated together with constipation. Therefore, this study was conducted to further observe the relationship between DII and E-DII and constipation in American adults.

**Methods:**

Data were extracted from the National Health and Nutrition Examination Survey (NHANES) for 12,400 adults aged 20 years and older between 2005 and 2010. DII and E-DII were obtained by employing data from the two 24-h dietary recall of the participants. Constipation was defined and categorized using the Bristol Stool Form Scale.

**Results:**

In the logistic regression model, the relationship between DII and E-DII and constipation remained positive after adjusting for confounding factors (odds ratio [*OR*] = 1.13; 95% confidence interval [*CI*]: 1.07–1.20 in DII logistic regression model III; odds ratio [*OR*] = 1.09; 95% confidence interval [*CI*]: 1.03–1.17 in E-DII logistic regression model III). Constipation was more common in quartile 4 (DII: 2.87—5.09; E-DII: 1.78—8.95) than in quartile 1 (DII: -5.11—0.25; E-DII: -2.60—0.11) (*OR* = 1.79, 95% *CI*: 1.30–2.47 in DII and *OR* = 1.75, 95% *CI*: 1.25–2.46 in E-DII for all participants; *OR* = 2.04, 95% *CI*: 1.39–3.00 in DII *OR* = 2.20, 95% *CI*: 1.39–3.47 in E-DII for males; *OR* = 1.86, 95% *CI*: 1.08–3.22 and *OR* = 1.80, 95% *CI*: 1.06–3.06 for females). These results were confirmed using multiple imputations.

**Conclusions:**

The findings of this study show that a high DII and E-DII were associated with an increased incidence of constipation among US adults.

**Supplementary Information:**

The online version contains supplementary material available at 10.1186/s12876-024-03307-7.

## Background

Constipation is a very common intestinal problem. Previous studies have indicated that the incidence of constipation in the population studied, according to the Rome IV diagnostic criteria, is in the range of 7.9–8.6% and that the prevalence is lower in males than that in females [1]. Constipation increases the social burden and reduces the living standards of an individual [2]. Moreover, individuals with constipation have an elevated risk of death [3]. Thus, the treatment of constipation has attracted considerable attention and individuals with constipation are increasingly looking to modify their diets to relieve constipation.

Dietary factors are closely related to constipation [4]. The inflammatory potential of a diet refers to its potential to cause inflammation [5]. As a person’s daily diet contains various food types and dietary nutrients, specific interactions between these substances may exist, and the cumulative effect may impact the level of inflammation in the body [6]. A diet may be pro-inflammatory or anti-inflammatory. A pro-inflammatory diet can promote the onset of disease and its exacerbation, including colorectal cancer, constipation, and metabolic syndrome [3, 7, 8]. In addition, a pro-inflammatory diet can increase the levels of inflammatory markers. The intake of fruits, vegetables, and seafood may decrease the inflammatory markers; however, the intake of high-carb and high-fat diets has been linked to higher levels of these inflammatory markers [9, 10]. The higher the dietary inflammatory index (DII) and score, the greater the pro-inflammatory effect; the lower the DII and energy-dietary inflammation index (E-DII) score, the greater the anti-inflammatory effect. The intake of fewer fruits and vegetables [11, 12], and low intake of dietary fibre, water, and energy can lead to constipation [13, 14]. Moreover, Tan et al. showed that chronic intestinal inflammation and constipation caused by high-fructose and high-fat foods led to disruption in intestinal microbiota and microbial metabolites [15].

DII is objective index to assess the inflammatory potential of the diet [6, 16]. A high DII diet induces an inflammatory response, whereas a low DII diet contributes to an increase in the anti-inflammatory effect. DII is positively correlated with dietary inflammatory markers and is commonly used to measure the level of diet-borne inflammation [17, 18]. An increase in DII causes an imbalance in the intestinal flora and leads to abnormal intestinal health [19, 20]. Mice fed a diet high in fat or fructose had elevated levels of bacteria associated with inflammation [15]. Regardless of the fat content in the diet, high sugar diet exposure can alter the composition of the gut microbiota and induce gastrointestinal inflammation.Solid high-fat, high-sugar diet intake induces more significant changes in the gut microbiota than liquid sucrose [21]. When these two diets are not digested properly, they may cause an imbalance in the intestinal flora, potentially leading to inflammation [15]. This may be related to the fact that a pro-inflammatory diet affects the gut microbiota, which in turn affects gastrointestinal motility by regulating the brain–gut axis, affecting stool excretion [22–24]. However, no previous studies extensively examined the relationship between DII and constipation. E-DII adjusted the energy intake on the basis of DII. The E-DII is calculated based on the intake per 1,000 kcal consumed. Increasing the energy-adjusted DII score was a better predictor of dietary inflammation [6]. The higher the score of E-DII, the more pro-inflammatory components in the diet [25]. Although the relationship between inflammation and constipation has been studied, no studies have evaluated the relationship between DII and E-DII and constipation at the same time [19, 20]. Therefore, this study investigated the relationship between DII and constipation and the relationship between E-DII and constipation in adults in the United States (US).

## Methods

### Study cohort

The National Health and Nutrition Examination Survey (NHANES) is a cross-sectional study survey of the non-hospitalized population in US by the National Centers for Health Statistics of the Centres for Disease Control and Prevention (CDC). This study was conducted to further observe the relationship between dietary inflammation and constipation among US adults. We analysed data from the NHANES database for three periods: 2005–2006, 2007–2008, and 2009–2010. Data from the study population were collected at 2-year intervals and a stratified multi-stage approach was used to obtain representative data. All participants signed consent forms for participation in this study. The Ethics Review Committee of the CDC approved the creation and use of NHANES data.

### Definition of constipation

We assessed constipation using the Bristol Stool Form Scale (BSFS). The constipation report was obtained through face-to-face interviews at the mobile examination centre (MEC). The BSFS has been used to define constipation in several studies because it is correlated with intestinal transit time [14, 26]. In the BSFS, stool consistency is categorised into seven types. Type 1 (separate hard lumps, like nuts) and Type 2 (sausage-like lumpy) are classified as constipation.

### Dietary inflammatory index

Accurate information regarding dietary intake was collected during two 24-h period. Each participant was interviewed twice, and dietary recalls were collected on both occasions [27]. The DII is a review of thousands of articles discussing the effects of 45 dietary components on inflammation [6]. A greater pro-inflammatory effect reflects a higher score, while a greater anti-inflammatory effect is represented by a lower score [6]. However, only 28 available dietary component results could be extracted from the NHANES database [28]. Previous studies have shown that using 28 dietary components for predictions did not affect the results [5]. In the NHANES, DII was calculated using 28 food parameters: energy, protein, total fat, fibre, cholesterol, monounsaturated fatty acids, saturated fat, polyunsaturated fatty acids, n-6 fatty acids, n-3 fatty acids, niacin, thiamine, riboflavin, vitamin B12, vitamin B6, vitamin C, vitamin A, vitamin E, Vitamin D, ferrum, magnesium, zinc, selenium, folic acid, beta-carotene, caffeine, alcohol, and carbohydrates. The specific DII calculation methods have been previously described [29–31]. E-DII scores were measured by calculating DII per 1000-kilocalorie consumption and employed the same scoring procedure [32]

### Covariates

The following variables were included in this study: ethnicity (non-Hispanic white, non-Hispanic black, or others); age (years) (< 45, 45–65, and ≥ 65); sex; marital status (single, married, or living with a partner); income-poverty ratio (%) (< 2, ≥ 2, or not recorded); education (< high school, high school, or > high school); alcohol consumption status (never, former, or current); smoking status (never, former, or current); physical activity (*MET*-min/week: < 500, ≥ 500, or not recorded); body mass index (*BMI*: < 25, 25–30, ≥ 30 kg/m^2^); depression; diabetes; hypertension; and intake of tea, coffee, moisture, plain water, tap water, bottled water, dietary fibre, and energy.

### Statistical analysis

We applied the weights suggested by the CDC. These weights consider sampling bias, resulting in more accurate information. Continuous variables are presented as median (Q1–Q3), whereas categorical variables are presented as percentage (95% *CI*).

The basic characteristics are shown for the study population (Tables 1 and 2). To clarify the relationship between DII and E-DII and constipation, we used three different logistic regression models. DII and E-DII are increased by one unit (one standard deviation), and Q1 was used as a reference to observe differences between the odds ratios (*ORs*) and 95% *CI* for Q2, Q3, and Q4 and the difference between the results for both sexes. Model I was not adjusted for covariates; Model II was adjusted for three confounders – age, sex, and ethnicity; and Model III was adjusted for age; sex; ethnicity; education; marital,smoking; BMI; income-poverty ratio; physical activity; depression; hypertension; tea; coffee; moisture; tap water; and bottled water.

**Table 1:** Population characteristics by DII, weighted

Variables	Overall	Quartile 1	Quartile 2	Quartile 3	Quartile 4	*P*-value
DII	1.5 (0.0,2.7)	-0.9(-1.7,-0.3)	1.1 (0.7,1.4)	2.3 (2.0,2.6)	3.5 (3.2,3.8)	< 0.0001
EDII	0.7 (0.0,1.6)	-0.4 (-0.7,-0.1)	0.5 (0.3,0.7)	1.3 (1.0,1.6)	2.4 (1.8,3.2)	< 0.0001
Tea intake (g/day)	0.0 (0.0,236.8)	0.0 (0.0,259.0)	0.0 (0.0,222.0)	0.0 (0.0,251.6)	0.0 (0.0,195.4)	0.0044
Coffee intake (g/day)	178.9 (0.0,488.4)	244.2 (0.0,569.8)	207.2 (0.0,510.6)	155.4 (0.0,460.0)	119.2 (0.0,406.9)	0.0025
Moisture intake (g/day)	2706.9 (2047.5,3590.8)	3319.4 (2632.2,4194.6)	2816.5 (2210.2,3613.5)	2483.7 (1923.1,3257.4)	2049.5 (1566.1,2717.4)	< 0.0001
Plain water intake (g/day)	740.6 (281.4,1422.0)	1056.1 (474.0,1718.2)	784.4 (311.1,1436.8)	651.8 (250.3,1296.1)	500.0 (133.3,1094.1)	< 0.0001
Tap water intake (g/day)	288.8 (0.0,925.8)	503.6 (7.4,1273.9)	288.8 (0.0,903.6)	266.6 (0.0,799.9)	118.4 (0.0,592.5)	< 0.0001
Bottled water intake (g/day)	0.0 (0.0,500.9)	0.0 (0.0,516.6)	0.0 (0.0,592.2)	0.0 (0.0,487.0)	0.0 (0.0,444.4)	0.1153
Fibre intake (g/day)	15.1 (10.6,20.8)	23.6 (19.4,28.8)	16.4 (13.4,19.9)	12.6 (10.1,15.4)	8.8 (6.6,11.2)	< 0.0001
Age (years)						0.0303
< 45	45.9 (43.9,47.9)	43.4 (39.6,47.2)	46.1 (43.2,49.0)	47.0 (44.0,50.0)	47.6 (45.1,50.2)	
≥ 45, < 65	36.6 (35.3,38.0)	38.6 (35.9,41.4)	37.6 (35.3,40.0)	36.3 (33.9,38.8)	33.4 (31.0,35.8)	
≥ 65	17.5 (16.2,18.8)	18.0 (15.5,20.7)	16.3 (14.6,18.2)	16.7 (15.0,18.6)	19.0 (17.4,20.7)	
Sex						< 0.0001
Female	51.7 (50.7,52.6)	39.0 (37.1,41.0)	46.1 (43.8,48.5)	57.4 (55.3,59.5)	67.9 (65.5,70.2)	
Male	48.3 (47.4,49.3)	61.0 (59.0,62.9)	53.9 (51.5,56.2)	42.6 (40.5,44.7)	32.1 (29.8,34.5)	
Ethnicity, %						< 0.0001
Non-Hispanic White	71.9 (67.9,75.6)	77.5 (73.6,81.0)	72.2 (67.9,76.1)	69.2 (64.4,73.7)	67.4 (62.1,72.3)	
Non-Hispanic Black	11.0 (9.2,13.1)	6.7 (5.3,8.5)	9.7 (7.9,11.9)	13.2 (10.8,16.0)	15.6 (12.9,18.7)	
Others	17.1 (14.5,20.1)	15.8 (13.1,19.0)	18.1 (15.4,21.2)	17.6 (14.5,21.1)	17.0 (13.4,21.3)	
Marital status, %						< 0.0001
Single	36.0 (33.9,38.1)	31.8 (28.9,34.9)	32.9 (29.6,36.4)	38.0 (35.4,40.6)	42.5 (39.8,45.3)	
Married or living with partner	64.0 (61.9,66.1)	68.2 (65.1,71.1)	67.1 (63.6,70.4)	62.0 (59.4,64.6)	57.5 (54.7,60.2)	
Family income-to-poverty ratio (PIR)						< 0.0001
< 2	30.7 (28.7,32.8)	22.5 (20.3,24.9)	27.8 (25.6,30.2)	32.2 (28.8,35.8)	42.7 (39.8,45.7)	
≥ 2	63.8 (61.5,66.0)	72.4 (69.7,74.9)	66.2 (63.4,68.8)	62.7 (58.6,66.5)	51.5 (48.3,54.6)	
Not recorded	5.5 (4.8,6.3)	5.1 (4.1,6.4)	6.0 (4.8,7.4)	5.1 (4.1,6.4)	5.8 (4.7,7.2)	
Education, %						< 0.0001
< High school	5.6 (4.8,6.5)	3.3 (2.6,4.2)	5.3 (4.3,6.5)	5.9 (4.9,7.0)	8.3 (7.0,9.9)	
High school	36.2 (34.0,38.5)	25.9 (23.4,28.5)	34.3 (31.4,37.4)	40.8 (38.0,43.6)	46.7 (43.6,49.9)	
> High school	58.2 (55.6,60.7)	70.8 (68.1,73.3)	60.4 (57.1,63.6)	53.3 (50.3,56.3)	44.9 (41.8,48.1)	
Smoking, %						< 0.0001
Never	52.9 (50.9,54.9)	56.2 (52.7,59.7)	53.0 (49.9,56.1)	54.8 (52.5,57.0)	46.7 (43.4,50.0)	
Former	25.3 (23.8,26.8)	30.3 (27.5,33.3)	26.4 (24.3,28.6)	21.1 (19.5,22.8)	22.1 (20.2,24.1)	
Now	21.8 (20.5,23.2)	13.4 (11.7,15.3)	20.6 (18.5,22.9)	24.1 (22.1,26.3)	31.2 (28.3,34.3)	
Alcohol, %						< 0.0001
Never	10.7 (9.5,12.1)	8.2 (6.7,9.9)	9.7 (8.4,11.2)	10.9 (8.9,13.4)	14.9 (13.3,16.7)	
Former	16.9 (15.5,18.4)	13.8 (11.9,15.9)	14.4 (12.5,16.4)	18.3 (16.4,20.3)	22.3 (20.1,24.6)	
Now	72.4 (70.3,74.4)	78.0 (75.7,80.1)	75.9 (73.1,78.5)	70.8 (67.9,73.6)	62.9 (60.0,65.7)	
Milk						< 0.0001
Often	41.9 (40.1,43.7)	51.5 (48.3,54.8)	44.8 (41.8,47.8)	37.2 (34.8,39.5)	31.5 (28.8,34.4)	
Sometimes	28.6 (27.5,29.8)	24.9 (22.5,27.5)	27.0 (24.5,29.7)	32.6 (30.6,34.6)	30.9 (28.2,33.6)	
Rarely	13.9 (13.0,15.0)	10.3 (8.8,11.9)	13.3 (11.4,15.4)	14.7 (12.9,16.7)	18.5 (16.6,20.4)	
Never	15.2 (14.3,16.3)	12.9 (11.2,14.8)	14.7 (12.9,16.7)	15.3 (13.6,17.1)	18.9 (16.8,21.0)	
Varied	0.3 (0.2,0.5)	0.4 (0.1,1.1)	0.2 (0.1,0.4)	0.2 (0.1,0.6)	0.3 (0.2,0.7)	
BMI (kg/m2)						< 0.0001
< 25	30.8 (29.1,32.7)	36.6 (33.3,40.1)	27.0 (24.4,29.7)	29.4 (27.0,32.0)	29.4 (26.3,32.7)	
≥ 25, < 30	33.8 (32.4,35.3)	34.5 (32.1,37.0)	36.7 (33.9,39.4)	33.6 (30.9,36.5)	29.9 (27.4,32.4)	
≥ 30	35.3 (33.7,37.0)	28.9 (26.0,31.9)	36.4 (33.3,39.6)	36.9 (34.4,39.4)	40.7 (37.6,43.9)	
Physical activity (MET-min/week), %						< 0.0001
< 500	21.7 (20.3,23.2)	19.3 (17.5,21.3)	22.1 (19.7,24.7)	24.6 (21.9,27.6)	21.1 (18.6,23.7)	
≥ 500	57.6 (55.6,59.5)	67.1 (63.9,70.1)	58.2 (55.6,60.8)	53.0 (49.7,56.3)	49.7 (46.9,52.5)	
Not recorded	20.7 (19.5,22.1)	13.6 (11.7,15.8)	19.7 (18.0,21.5)	22.4 (20.4,24.4)	29.2 (26.7,31.9)	
Depression symptoms, %						< 0.0001
No	92.4 (91.5,93.2)	96.3 (95.5,97.0)	93.6 (92.3,94.7)	91.8 (90.2,93.2)	86.7 (84.5,88.7)	
Yes	7.6 (6.8,8.5)	3.7 (3.0,4.5)	6.4 (5.3,7.7)	8.2 (6.8,9.8)	13.3 (11.3,15.5)	
Diabetes, %						0.0001
No	87.3 (86.2,88.3)	89.5 (88.1,90.8)	87.6 (85.9,89.2)	86.8 (85.0,88.4)	84.6 (82.5,86.4)	
Yes	12.7 (11.7,13.8)	10.5 (9.2,11.9)	12.4 (10.8,14.1)	13.2 (11.6,15.0)	15.4 (13.6,17.5)	
Hypertension, %						0.0019
No	63.1 (61.3,64.9)	66.3 (63.3,69.1)	62.7 (59.5,65.8)	63.5 (60.9,65.9)	59.4 (56.5,62.1)	
Yes	36.9 (35.1,38.7)	33.7 (30.9,36.7)	37.3 (34.2,40.5)	36.5 (34.1,39.1)	40.6 (37.9,43.5)	
Constipation, %						< 0.0001
No	92.9 (92.3,93.4)	95.4 (94.4,96.2)	94.3 (93.1,95.3)	92.3 (91.0,93.4)	88.8 (87.3,90.2)	
Yes	7.1 (6.6,7.7)	4.6 (3.8,5.6)	5.7 (4.7,6.9)	7.7 (6.6,9.0)	11.2 (9.8,12.7)	

Continuous variables are expressed as weighted median (Q1-Q3) and categorical variables are expressed as percentage (95% *CI*)

DII quartile ranges: Quartile 1 = -5.11—0.25; Quartile 2 = 1.72—2.87; Quartile 3 = 1.72—2.87,Quartile 4 = 2.87—5.09

*Abbreviations*: *NHANES* National health and nutrition examination survey, *DII* Dietary inflammatory index, *E-DII* Energy-adjusted dietary inflammatory index, *BMI* Body mass index

**Table 2:** Population characteristics by E-DII, weighted

Variables	Quartile 1	Quartile 2	Quartile 3	Quartile 4	*P*-value
DII	-0.9 (-1.7,-0.3)	1.1 (0.7,1.5)	2.3 (1.9,2.8)	3.4 (3.0,3.8)	< 0.0001
EDII	-0.4 (-0.7,-0.1)	0.5 (0.3,0.7)	1.3 (1.1,1.5)	2.5 (2.1,3.3)	< 0.0001
Tea intake (g/day)	0.0 (0.0,259.0)	0.0 (0.0,236.8)	0.0 (0.0,244.2)	0.0 (0.0,185.0)	0.0008
Coffee intake (g/day)	244.2 (0.0,577.2)	178.8 (0.0,503.2)	163.0 (0.0,474.4)	125.8 (0.0,384.8)	0.0005
Moisture intake (g/day)	3344.2 (2645.1,4206.8)	2885.6 (2272.6,3693.6)	2441.0 (1923.1,3222.9)	1975.1 (1496.8,2599.6)	< 0.0001
Plain water intake (g/day)	1048.1 (474.0,1717.5)	740.4 (288.8,1399.1)	614.7 (228.7,1244.2)	592.5 (207.4,1185.0)	< 0.0001
Tap water intake (g/day)	503.6 (22.2,1273.1)	288.8 (0.0,896.2)	236.8 (0.0,777.7)	148.1 (0.0,622.1)	< 0.0001
Bottled water intake (g/day)	0.0 (0.0,518.4)	0.0 (0.0,518.1)	0.0 (0.0,474.0)	0.0 (0.0,500.9)	0.3767
Fibre intake (g/day)	23.6 (19.4,28.9)	16.3 (13.4,19.7)	12.4 (9.9,15.2)	8.8 (6.5,11.2)	< 0.0001
Age (years)					< 0.0001
< 45	43.5 (39.7,47.4)	49.0 (46.3,51.7)	47.7 (44.8,50.6)	43.2 (40.7,45.7)	
≥ 45, < 65	38.7 (36.0,41.5)	37.3 (34.9,39.7)	36.2 (33.8,38.6)	33.7 (31.2,36.2)	
≥ 65	17.8 (15.3,20.5)	13.7 (12.2,15.4)	16.2 (14.2,18.3)	23.1 (21.5,24.8)	
Sex					< 0.0001
Female	38.1 (36.2,40.0)	41.5 (38.9,44.1)	57.7 (55.8,59.5)	75.1 (73.2,76.9)	
Male	61.9 (60.0,63.8)	58.5 (55.9,61.1)	42.3 (40.5,44.2)	24.9 (23.1,26.8)	
Ethnicity, %					< 0.0001
Non-Hispanic White	77.5 (73.6,81.0)	72.5 (68.3,76.3)	69.6 (64.9,73.9)	66.5 (61.1,71.5)	
Non-Hispanic Black	6.8 (5.4,8.6)	10.1 (8.3,12.2)	12.7 (10.4,15.5)	15.7 (12.9,18.8)	
Others	15.7 (13.0,18.8)	17.4 (14.8,20.4)	17.7 (14.7,21.1)	17.9 (14.2,22.3)	
Marital status, %					< 0.0001
Single	31.7 (28.8,34.8)	33.5 (30.3,36.8)	37.0 (33.8,40.4)	43.3 (40.9,45.7)	
Married or living with partner	68.3 (65.2,71.2)	66.5 (63.2,69.7)	63.0 (59.6,66.2)	56.7 (54.3,59.1)	
Family income-to-poverty ratio (PIR)					< 0.0001
< 2	22.4 (20.2,24.8)	27.9 (25.4,30.5)	32.8 (29.8,35.9)	42.6 (39.7,45.6)	
≥ 2	72.6 (69.9,75.1)	66.7 (64.1,69.2)	61.4 (57.9,64.7)	51.5 (48.2,54.8)	
Not recorded	5.0 (4.0,6.2)	5.4 (4.3,6.9)	5.9 (4.7,7.4)	5.9 (4.8,7.2)	
Education, %					< 0.0001
< High school	3.3 (2.6,4.2)	4.6 (3.7,5.5)	5.6 (4.7,6.7)	9.7 (8.0,11.6)	
High school	26.4 (23.9,29.1)	34.2 (31.2,37.3)	42.4 (39.5,45.4)	44.7 (41.4,48.0)	
> High school	70.3 (67.6,72.9)	61.3 (57.9,64.5)	51.9 (48.8,55.0)	45.7 (42.5,48.8)	
Smoking, %					< 0.0001
Never	56.0 (52.7,59.3)	52.2 (49.2,55.2)	53.4 (50.7,56.1)	49.2 (45.9,52.5)	
Former	30.1 (27.4,33.0)	25.7 (23.2,28.3)	21.6 (19.9,23.4)	22.6 (20.5,24.9)	
Now	13.9 (12.1,15.8)	22.2 (20.2,24.2)	25.0 (22.6,27.5)	28.2 (25.2,31.4)	
Alcohol, %					< 0.0001
Never	8.1 (6.6,9.8)	8.6 (7.2,10.2)	10.9 (9.1,13.0)	16.7 (14.7,18.9)	
Former	13.8 (11.9,16.0)	14.2 (12.3,16.4)	18.6 (16.4,20.9)	22.3 (19.9,25.0)	
Now	78.1 (75.8,80.2)	77.2 (74.2,79.9)	70.5 (68.0,73.0)	61.0 (58.2,63.8)	
Milk					< 0.0001
Often	51.9 (48.7,55.2)	42.3 (39.4,45.3)	37.0 (34.4,39.6)	33.8 (31.0,36.7)	
Sometimes	24.4 (22.0,27.1)	29.3 (26.9,31.7)	32.1 (29.8,34.5)	29.5 (26.9,32.2)	
Rarely	10.4 (9.0,12.1)	13.5 (11.6,15.6)	15.1 (13.3,17.0)	17.8 (16.0,19.7)	
Never	12.8 (11.1,14.8)	14.7 (12.8,16.8)	15.6 (13.7,17.7)	18.7 (17.0,20.5)	
Varied	0.4 (0.1,1.1)	0.2 (0.1,0.4)	0.3 (0.2,0.5)	0.3 (0.1,0.7)	
BMI (kg/m2)					< 0.0001
< 25	36.2 (32.9,39.7)	27.6 (25.0,30.5)	29.7 (27.1,32.3)	28.9 (26.4,31.6)	
≥ 25, < 30	35.1 (32.6,37.6)	35.7 (33.4,38.0)	32.4 (30.2,34.8)	31.6 (28.7,34.5)	
≥ 30	28.7 (26.0,31.7)	36.7 (33.7,39.8)	37.9 (35.6,40.3)	39.5 (36.6,42.5)	
Physical activity (MET-min/week), %					< 0.0001
< 500	19.4 (17.6,21.3)	22.4 (19.9,25.0)	23.9 (20.7,27.4)	21.4 (19.3,23.7)	
≥ 500	67.0 (64.0,69.9)	58.9 (56.2,61.5)	53.4 (49.9,57.0)	48.2 (45.8,50.7)	
Not recorded	13.6 (11.7,15.8)	18.8 (16.9,20.7)	22.7 (20.7,24.7)	30.3 (28.0,32.8)	
Depression symptoms, %					< 0.0001
No	96.3 (95.3,97.0)	93.5 (92.1,94.7)	91.3 (89.7,92.7)	87.2 (85.1,89.0)	
Yes	3.7 (3.0,4.7)	6.5 (5.3,7.9)	8.7 (7.3,10.3)	12.8 (11.0,14.9)	
Diabetes, %					< 0.0001
No	89.6 (88.2,90.8)	88.9 (87.3,90.4)	86.3 (84.5,87.9)	83.4 (81.3,85.2)	
Yes	10.4 (9.2,11.8)	11.1 (9.6,12.7)	13.7 (12.1,15.5)	16.6 (14.8,18.7)	
Hypertension, %					0.0001
No	66.1 (63.1,69.0)	64.1 (60.9,67.2)	63.2 (60.6,65.7)	58.0 (55.3,60.6)	
Yes	33.9 (31.0,36.9)	35.9 (32.8,39.1)	36.8 (34.3,39.4)	42.0 (39.4,44.7)	
Constipation					< 0.0001
No	95.4 (94.4,96.2)	94.6 (93.4,95.6)	91.7 (90.1,93.0)	88.9 (87.2,90.5)	
Yes	4.6 (3.8,5.6)	5.4 (4.4,6.6)	8.3 (7.0,9.9)	11.1 (9.5,12.8)	

Continuous variables are expressed as weighted median (Q1-Q3) and categorical variables are expressed as percentage (95% *CI*)

E-DII quartile ranges: Quartile 1 = -2.60—0.11; Quartile 2 = 0.11—0.86; Quartile 3 = 0.86—1.78, Quartile 4 = 1.78—8.95

*Abbreviations*: *NHANES* National health and nutrition examination survey, *DII* Dietary inflammatory index, *E-DII* Energy-adjusted dietary inflammatory index, *BMI* Body mass index

We calculated the value of the trend test in the models and the *OR* (95% *CI*) for the association between each continuous variable and constipation. We then examined the relationship between each variable and constipation using univariate analysis. Furthermore, to observe the relationships more intuitively, we examined the trends in both constipation and continuous variables using smooth curve fitting. We used the multiple imputation method to construct a logistic regression model. We used *R* and the Empower package (The *R* Foundation; http://www.r-project.org; version 4.2.0) for the statistical analyses, and a *P *value < 0.05 was considered as statistically significant.

## Results

### Baseline characteristics of the participants

**Fig. 1:**
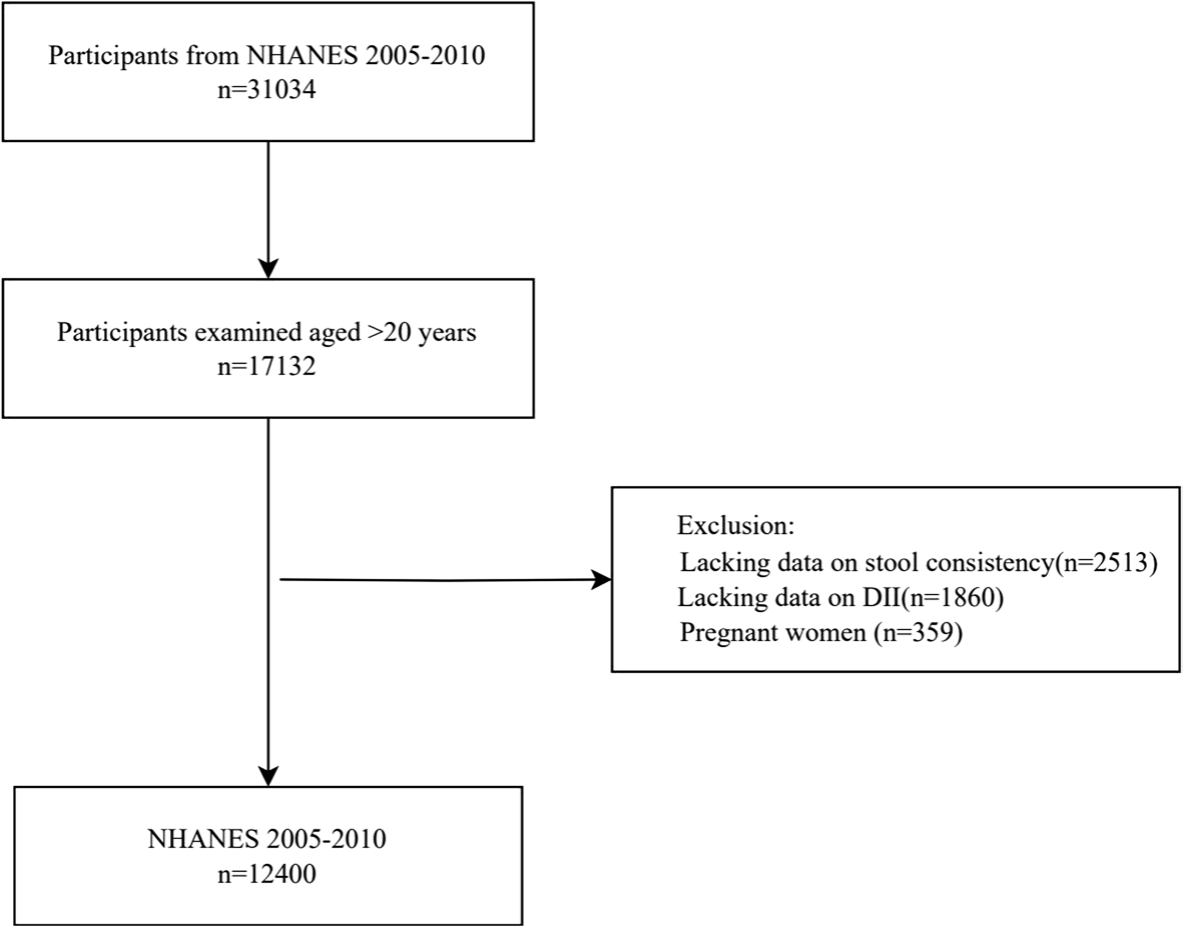
Flow diagram of selection. Notes: The NHANES database had a total of 31034 participants from 2005–2010. Then, we screened out people who were 20 years or older (*n* = 17,132). Next, the following participants were further excluded: lacking data on stool consistency (*n* = 2513), lacking data on DII (*n* = 1860), and pregnant women (*n* = 359). Ultimately, data on 12400 participants were retained. Abbreviations: NHANES, National Health and Nutrition Examination Survey; DII, dietary inflammatory index

Originally, 31,034 participants were drawn from the survey population. After excluding the participants under the age of 20 years, 17,132 were finally included in the study. After further screening, we excluded the participants without data on stool consistency (*n* = 2513) and DII (*n* = 1860). Pregnant females (*n* = 359) were also excluded because they are prone to constipation due to changes in the gastrointestinal tract [33]. Since including pregnant females would reduce the reliability of the findings, most studies that reported constipation in the literature excluded them [14, 34]. Ultimately, 12,400 individuals were retained for further analysis. The flowchart of the participant selection is shown in Fig. 1. Tables 1 and 2 list the characteristics of the weighted population; 48.3% of the participants were males. In total, 7.1% of the participants had constipation. The results showed that dietary intake decreased gradually as DII and E-DII increased.

### DII and constipation

From the smooth curve-fitting graph in Supplementary Figs. 1 and 2, DII and E-DII and constipation can be intuitively understood to possess a non-linear relationship. This relationship was further explored using three multiple-regression models (Tables 3 and 4). Logistic regression showed a positive correlation between DII and constipation (1.23, 1.16–1.30 in Model I; 1.13, 1.07–1.20 in Model II; and 1.13, 1.07–1.20 in Model III). There are similar trends in E-DII and constipation (1.22, 1.17–1.29 in Model I; 1.11, 1.05–1.17 in Model II; and 1.09, 1.03–1.17 in Model III). In Model III, the overall prevalence in Q4 was significantly higher than that in Q1 (DII regression model: 1.79, 1.30–2.47; *P* value for trend < 0.001; E-DII regression model: 1.75, 1.25–2.46; *P* value for trend = 0.001). In model III, this trend was observed in both sexes (DII regression model: 2.04, 1.39–3.00; *P* value for trend = 0.001 for males and 1.86, 1.08–3.22; *P* value for trend = 0.013 for females; E-DII regression model: 2.20, 1.39–3.47; *P* value for trend = 0.003 for males and 1.80, 1.06–3.06; *P* value for trend = 0.018 for females). This correlation was also observed after multiple imputations (Supplementary Tables 1 and 2).

**Table 3:** Association of DII with constipation, weighted

Exposure	Model I	Model II	Model III
DII			
Number of cases	12400	12390	12258
Continues	1.23(1.16,1.30)	1.13(1.07,1.20)	1.14(1.08,1.22)
Per-SD increase	1.45(1.31,1.61)	1.25(1.13,1.39)	1.28(1.14,1.42)
Q1	Ref	Ref	Ref
Q2	1.23(0.91,1.69)	1.09(0.79,1.51)	1.13(0.82,1.55)
Q3	1.71(1.31,2.83)	1.32(1.00,1.75)	1.39(1.06,1.85)
Q4	2.59(1.98,3.39)	1.77(1.32,2.40)	1.88(1.39,2.55)
P for trend	< 0.001	< 0.001	0.001
Men			
Number of cases	6139	6135	6060
Continues	1.26(1.12,1.42)	1.20(1.07,1.35)	1.17(1.05,1.31)
Per-SD increase	1.52(1.22,1.89)	1.39(1.13,1.73)	1.34(1.09,1.65)
Q1	Ref	Ref	Ref
Q2	1.29(0.71,2.37)	1.19(0.66,2.16)	1.22(0.67,2.23)
Q3	1.67(0.99,2.83)	1.45(0.87,2.41)	1.41(0.83,2.39)
Q4	3.04(1.72,5.35)	2.47(1.43,4.29)	2.30(1.32,4.04)
P for trend	< 0.001	0.002	0.005
Female			
Number of cases	6261	6255	6198
Continues	1.14(1.06,1.22)	1.10(1.03,1.18)	1.13(1.05,1.22)
Per-SD increase	1.24(1.11,1.39)	1.18(1.05,1.33)	1.24(1.10,1.40)
Q1	Ref	Ref	Ref
Q2	1.13(0.82,1.56)	1.07(0.78,1.47)	1.14(0.82,1.60)
Q3	1.29(0.96,1.74)	1.17(0.86,1.59)	1.26(0.88,1.80)
Q4	1.77(1.36,2.31)	1.54(1.16,2.06)	1.75(1.20,2.56)
* P* for trend	< 0.001	0.005	0.007

Model I: no covariates were adjusted

Model II: adjusted for age; sex; ethnicity; education

Model III: adjusted for age; sex; ethnicity; education; marital,smoking; BMI; income-poverty ratio; physical activity; depression; hypertension, tea, coffee, moisture, tap water, bottled water

DII quartile ranges: Quartile 1 = -5.11—0.25; Quartile 2 = 1.72—2.87; Quartile 3 = 1.72—2.87,Quartile 4 = 2.87—5.09

E-DII quartile ranges: Quartile 1 = -2.60—0.11; Quartile 2 = 0.11—0.86; Quartile 3 = 0.86—1.78, Quartile 4 = 1.78—8.95

*Abbreviations*: *NHANES* National health and nutrition examination survey, *DII* Dietary inflammatory index, *E-DII* Energy-adjusted dietary inflammatory index, *BMI* Body mass index

**Table 4:** Association of E-DII with constipation, weighted

Exposure	Model I	Model II	Model III
E-DII			
Number of cases	12400	12390	12258
Continues	1.22(1.17,1.29)	1.11(1.05,1.17)	1.10(1.03,1.17)
Per-SD increase	1.23(1.14,1.32)	1.16(1.07,1.25)	1.15(1.04,1.26)
Q1	Ref	Ref	Ref
Q2	1.17(0.85,1.63)	1.07(0.77,1.48)	1.10(0.79,1.53)
Q3	1.87(1.40,2.50)	1.44(1.06,1.94)	1.51(1.13,2.03)
Q4	2.57(1.93,3.41)	1.69(1.23,2.31)	1.83(1.29,2.59)
for trend	< 0.001	< 0.001	0.001
Men			
Number of cases	6139	6135	6051
Continues	1.34(1.18,1.53)	1.21(1.10,1.32)	1.13(1.04,1.24)
Per-SD increase	1.53(1.28,1.84)	1.31(1.15,1.49)	1.20(1.05,1.36)
Q1	Ref	Ref	Ref
Q2	1.14(0.69,1.90)	0.99(0.65,1.50)	1.27(0.77,2.11)
Q3	2.05(1.28,3.29)	1.67(1.22,2.29)	1.87(1.26,2.79)
Q4	3.01(1.49,6.09)	2.19(1.45,3.31)	2.31(1.43,3.74)
P for trend	< 0.001	0.004	0.003
Female			
Number of cases	6261	6255	6207
Continues	1.10(1.05,1.17)	1.16(1.07,1.25)	1.13(1.00,1.27)
Per-SD increase	1.15(1.07,1.25)	1.24(1.10,1.39)	1.19(1.01,1.41)
Q1	Ref	Ref	Ref
Q2	1.13(0.75,1.71)	1.19(0.79,1.80)	0.94(0.60,1.46)
Q3	1.41(0.97,2.49)	1.68(1.08,2.62)	1.42(0.84,2.40)
Q4	1.69(1.19,2.41)	2.18(1.45,3.28)	1.86(1.09,3.19)
* P* for trend	< 0.001	0.021	0.014

Model I: no covariates were adjusted

Model II: adjusted for age; sex; ethnicity; education

Model III: adjusted for age; sex; ethnicity; education; marital,smoking; BMI; income-poverty ratio; physical activity; depression; hypertension, tea, coffee, moisture, tap water, bottled water

DII quartile ranges: Quartile 1 = -5.11—0.25; Quartile 2 = 1.72—2.87; Quartile 3 = 1.72—2.87,Quartile 4 = 2.87—5.09

E-DII quartile ranges: Quartile 1 = -2.60—0.11; Quartile 2 = 0.11—0.86; Quartile 3 = 0.86—1.78, Quartile 4 = 1.78—8.95

*Abbreviations*: *NHANES* National health and nutrition examination survey, *DII* Dietary inflammatory index, *E-DII* Energy-adjusted dietary inflammatory index, *BMI* Body mass index

### Univariate analysis

The crude associations between constipation and demographic characteristics, smoking, alcohol consumption, physical activity, depression, diabetes, hypertension, and dietary intake are presented in Table 5. Several factors, including depression, non-Hispanic black ethnicity, and other ethnicities, influence the increased incidence of constipation. However, the incidence of constipation is reduced in males; it is also reduced in the people being married or living with a partner, with high income–poverty ratio, ≥ high school education, smoking, drinking, *BMI* ≥ 25 kg/m^2^, physical activity ≥ 500 MET-min/week, diabetes, and hypertension.

**Table 5:** Crude association of constipation with demographics, smoking, alcohol, physical activity, depression, diabetes, hypertension, and dietary intake

Variable	N	% (95%CI)	OR (95%CI)	*P*-value
Age (years)				
< 45	4957	7.74 (6.68,8.79)	Ref	
≥ 45, < 65	4293	6.38 (5.38,7.38)	0.81 (0.65, 1.02)	0.0808
≥ 65	3150	7.00 (5.93,8.07)	0.90 (0.70, 1.15)	0.3979
Sex				
Female	6261	9.82 (9.06,10.58)	Ref	
Male	6139	4.21 (3.37,5.05)	0.40 (0.32, 0.51)	< 0.0001
Ethnicity, %				
Non-Hispanic White	6344	6.26 (5.53,6.99)	Ref	
Non-Hispanic Black	2433	10.41 (8.52,12.31)	1.74 (1.37, 2.21)	< 0.0001
Others	3623	8.55 (6.83,10.27)	1.40 (1.06, 1.86)	0.0237
Marital status, %				
Single	4739	8.12 (7.01,9.23)	Ref	
Married or living with partner	7654	6.55 (5.83,7.26)	0.79 (0.64, 0.97)	0.0322
Family income-to-poverty ratio (PIR)				
< 2	5168	9.01 (7.79,10.22)	Ref	
≥ 2	6364	6.13 (5.40,6.86)	0.66 (0.54, 0.80)	0.0001
Not recorded	868	7.87 (5.28,10.46)	0.86 (0.58, 1.29)	0.4746
Education, %				
< High school	1395	10.43 (7.81,13.05)	Ref	
High school	4918	8.83 (7.66,10.00)	0.83 (0.60, 1.15)	0.2746
> High school	6077	5.71 (5.04,6.37)	0.52 (0.38, 0.71)	0.0002
Smoking, %				
Never	6491	7.81 (7.12,8.50)	Ref	
Former	3264	6.05 (5.00,7.09)	0.76 (0.61, 0.94)	0.0159
Now	2643	6.64 (5.23,8.06)	0.84 (0.66, 1.06)	0.1559
Alcohol, %				
Never	1622	10.68 (8.14,13.22)	Ref	
Former	2546	8.31 (7.12,9.49)	0.76 (0.55, 1.05)	0.105
Now	8218	6.31 (5.58,7.03)	0.56 (0.41, 0.77)	0.0008
BMI (kg/m2)				
< 25	3443	9.04 (7.70,10.39)	Ref	
≥ 25, < 30	4249	6.98 (6.00,7.97)	0.76 (0.60, 0.96)	0.0252
≥ 30	4603	5.48 (4.63,6.33)	0.58 (0.46, 0.75)	0.0001
Physical activity (MET-min/week), %				
< 500	2461	7.24 (5.70,8.79)	Ref	
≥ 500	6647	6.35 (5.76,6.93)	0.87 (0.68, 1.11)	0.2639
Not recorded	3292	9.08 (7.72,10.45)	1.28 (0.94, 1.74)	0.1221
Depression symptoms, %				
No	11319	6.65 (6.14,7.16)	Ref	
Yes	1068	12.63 (9.03,16.24)	2.03 (1.45, 2.84)	0.0001
Diabetes, %				
No	10160	7.23 (6.62,7.85)	Ref	
Yes	2240	6.28 (5.09,7.47)	0.86 (0.69, 1.07) 0.1903	0.1903
Hypertension, %				
No	7088	7.32 (6.54,8.11)	Ref	
Yes	5309	6.75 (6.00,7.49)	0.92 (0.77, 1.08)	0.313
Milk				
Often	5138	7.69 (6.69,8.69)	Ref	
Sometimes	3482	5.47 (4.59,6.36)	0.69 (0.55, 0.88)	0.005
Rarely	1761	7.06 (5.14,8.98)	0.91 (0.64, 1.30)	0.6132
Never	1975	8.55 (6.73,10.37)	1.12 (0.84, 1.50)	0.4339
Varied	44	11.36 (-3.30,26.01)	1.54 (0.35, 6.70)	0.5692
Tea intake (g/day)	12398	7.11 (6.55,7.66)	1.00 (1.00, 1.00)	0.7148
Coffee intake (g/day)	12398	7.11 (6.55,7.66)	1.00 (1.00, 1.00)	0.0015
Moisture intake (g/day)	12400	7.11 (6.55,7.66)	1.00 (1.00, 1.00)	< 0.0001
Tap water intake (g/day)	12400	7.11 (6.55,7.66)	1.00 (1.00, 1.00)	0.0004
Bottled water intake (g/day)	12400	7.11 (6.55,7.66)	1.00 (1.00, 1.00)	0.0004
Plain water intake (g/day)	12400	7.11 (6.55,7.66)	1.00 (1.00, 1.00)	< 0.0001
4Fibre intake (g/day)	12400	7.11 (6.55,7.66)	0.96 (0.95, 0.97)	< 0.0001
1Energy intake (kcal/day)	12400	7.11 (6.55,7.66)	1.00 (1.00, 1.00)	< 0.0001

*Abbreviations*: *BMI* Body mass index, *n* Number of observations

## Discussion

This study extensively analysed the relationship between changes in DII and E-DII and the incidence of constipation. The analysis of 12,400 participants concluded that DII is positively associated with the incidence of constipation. Both DII and E-DII in the highest quartile significantly increased the incidence of constipation compared to those in the lowest quartile.

The effect of DII and E-DII on the gastrointestinal system have been examined in many studies. A previous case–control study [35] showed that the pro-inflammatory effect of diet on colorectal adenomas was non-significant (1.07; 0.97–1.19; *P* = 0.18). An anti-inflammatory diet has been reported to increase the frequency of faecal excretion and the amount and variety of gut microbiota [19]. A prospective study of patients aged 20–40 years with constipation [36] showed that, after 4 weeks of oral administration of lactis V9, the constipation symptoms improved, the anti-inflammatory cytokines increased, and the pro-inflammatory cytokines decreased. In a case–control study, a one-unit increase in DII was associated with a 10% increase in the likelihood of colorectal cancer and a 65% increase in the fourth quantile compared with the first quantile in a logistic regression model adjusted for multiple latent variables [37]. In another study, the DII score in the diet was divided into four groups from low to high according to the quartile, demonstrating that more stool passed in groups with lower scores [38]. The results of these studies demonstrate that DII is correlated with gastrointestinal diseases.

Several studies have shown a relation between dietary inflammatory potential and constipation. The Mediterranean diet pattern, which is characterized by high coarse grains and high fruit intake, is considered an anti-inflammatory diet [39, 40]. A study [41] of six Mediterranean countries showed a significant inverse association between the Mediterranean Diet Quality Index and functional constipation in teenagers and children. Herbs and spices are also part of the anti-inflammatory diet pattern [42]; for instance, ginger positively affects constipation [43, 44]. In a study investigating constipation relief, oral Chinese medicines containing ingredients such as plantains and sesame seeds were as effective as lactulose [45]. A previous cross-sectional study [3] showed that elevated DII is associated with constipation. After adjusting for various confounding factors, the results of that study revealed a relationship between the fourth-class DII group and constipation. When the group with the lowest DII value was used as the reference group, the effect values and confidence intervals of the second, third, and fourth groups in the study were 1.208 (0.938–1.555), 1.305 (1.018–1.675), and 1.671 (1.332–2.097), respectively.

DII and constipation showed a non-linear relationship in our study. After adjusting for multiple factors, the regression analysis results imply that the incidence of constipation increases with the increase in DII and E-DII, regardless of whether DII and E-DII were continuous or categorical variables. In the regression models, the incidence of constipation was highest in the highest group of the fourth class; the results were the same for both males and females. Therefore, DII and E-DII can facilitate the identification of people who are prone to constipation. This may be attributed the fact that an increase in DII and E-DII can increase stool hardness, leading to constipation. Combining the findings of this study with previous findings, it can be concluded that the increase in DII and E-DII aggravates the dysregulation of intestinal flora and increases stool stiffness, thus causing constipation.

This study findings can be used as a reference for clinical research and the development of public health policies. While this study cannot prove causality, the results suggest that nutritionists and dieticians can recommend the increase in the intake of anti-inflammatory diets to prevent constipation. In addition, this study expands the existing clinical research on DII and E-DII and constipation.

Several mechanisms may explain the effect of DII and E-DII on constipation. A cross-sectional study [20] showed that the types and levels of gut microbes had been linked to changes in inflammatory cytokines caused by diet. A high-fat diet is pro-inflammatory [46]. Transplanting microorganisms from mice on a high-fat diet significantly increased intestinal penetration of CD3^+^ T cells and macrophages, promoting an intestinal microbial imbalance [47]. In addition, dysbiosis of the gut microbiota is associated with constipation [48]. Gastrointestinal microbes affect gastrointestinal movement, food digestion, and absorption by regulating the brain–gut axis [49, 50], which is the interaction between the brain and gut. This two-way feedback pathway plays an important role in the homeostasis of the gastrointestinal tract and central nervous system, including a variety of mechanisms of the nervous, endocrine, and immune systems [51]. These mechanisms can regulate physiological processes such as immune and inflammatory responses, as well as visceral pain, neurobehavior, intestinal barrier function, and intestinal movement, thereby affecting intestinal health [23]. When the brain–gut axis is tense or unstable, abnormal intestinal dynamics may occur, leading to the retention of food in the gut for a longer time, thus promoting the growth and reproduction of harmful bacteria and causing intestinal inflammation [52]. Intestinal inflammation can cause damage to the intestinal wall and may also affect the intestinal mucosal barrier and the balance of intestinal flora to affect intestinal peristalsis and defecation function, leading to constipation [15]. In addition, a disorder of the brain–gut axis may also lead to an imbalance of intestinal hormones, such as the abnormal secretion of gastrin, enterocapsin, and 5-hydroxytrytamine. These hormones play an important role in regulating intestinal peristalsis and defecation, further aggravating constipation [53].

This strengths of this study lie in the use of data from the NHANES, and its comprehensiveness allowed us to account for a wide range of potential confounders; thus, increasing the validity of our findings. Second, to avoid the diminished efficacy and bias of statistical tests associated with the direct exclusion of missing values, we used multiple interpolation and sensitivity analyses to calculate missing data. The use of the E-DII to assess the inflammatory potential of the diet strengthened our exposure assessment. In addition, this study had many participants and used weighted analyses from a representative population from the US.

Despite the strengths of our study, some limitations should be acknowledged. First, the cross-sectional nature of the NHANES data precludes the establishment of causality; prospective studies are needed to confirm the temporal relationship between the dietary inflammatory potential and constipation. Secondly, as dietary intake information was obtained based on participants' recall ability, there may be some degree of measurement error and recall bias in the self-reported dietary data and constipation assessment. Third, although the DII is still used as reported in many studies [5, 54–57], the methods for detecting potential dietary inflammation are constantly being updated, and future research needs to incorporate various dietary assessments to provide a more comprehensive assessment of long-term dietary inflammatory potential. Finally, our analysis focused on a limited number of covariates, and the potential influence of other unmeasured factors, such as medication use or specific medical conditions, cannot be ruled out.

Although we explored the relationship between DII, E-DII, and constipation, many directions are worth studying in this field in the future. First, to study the effects of DII and E-DII on constipation in chronic diseases such as diabetes, hypertension, and heart disease, as well as the elderly and long-term bedridden patients, these studies are crucial to highlight the significance of employing a healthy diet to regulate constipation in these specific populations. Second, more prospective studies of the relationship between dietary intake and constipation are needed to determine a possible causal relationship. Third, the inclusion of intestinal flora and intestinal inflammation in the relevant studies of constipation is helpful to discover the internal mechanism of constipation.

## Conclusions

The results of this study confirm that the prevalence of constipation in the population is positively correlated with DII, further showing that increased intake of pro-inflammatory diets may increase the incidence of constipation. This may be related to the fact that a pro-inflammatory diet affects the gut microbiota, which in turn affects gastrointestinal motility by regulating the brain–gut axis and affecting stool excretion [23, 47, 51]. The results were also consistent between the sexes. The findings of this study could facilitate the treatment of constipation. However, more prospective studies are required to trace the specific relationship and underlying mechanisms.

### Supplementary Information

Supplementary Material 1: Supplementary Figure 1. Association between DII and constipation. Supplementary Figure 2. Association between E-DII and constipation. Supplementary Table 1. Association of DII with constipation after imputation, weighted. Supplementary Table 2. Association of E-DII with constipation after imputation, weighted. Supplementary Table 3. Table Normal test.

## Data Availability

All the data used in this study are from the open database of NHANES (http://www.cdc.gov/nchs/nhanes.htm).

## Abbreviations

BMI: Body mass index
BSFS: Bristol Stool Form Scale
CDC: Centres for Disease Control and Prevention
CI: Confidence interval
DII: Dietary inflammation index
E-DII: Energy-dietary inflammation index
MEC: Mobile examination centre
NHANES: National Health and Nutrition Examination Survey
OR: Odds ratio
