# Supplementary material for: Association between dietary inflammatory index and energy-adjusted dietary inflammatory index and constipation in US adults
Source: BMC Gastroenterol. 2024 Jul 25;24:235. doi: 10.1186/s12876-024-03307-7 (PMC11282795; doi:10.1186/s12876-024-03307-7)
Supplement: Supplementary file 1 — Supplementary Material 1: Supplementary Figure 1. Association between DII and constipation. Supplementary Figure 2. Association between E-DII and constipation. Supplementary Table 1. Association of DII with constipation after imputation, weighted. Supplementary Table 2. Association of E-DII with constipation after imputation, weighted. Supplementary Table 3. Table Normal test. [file 12876_2024_3307_MOESM1_ESM.docx]

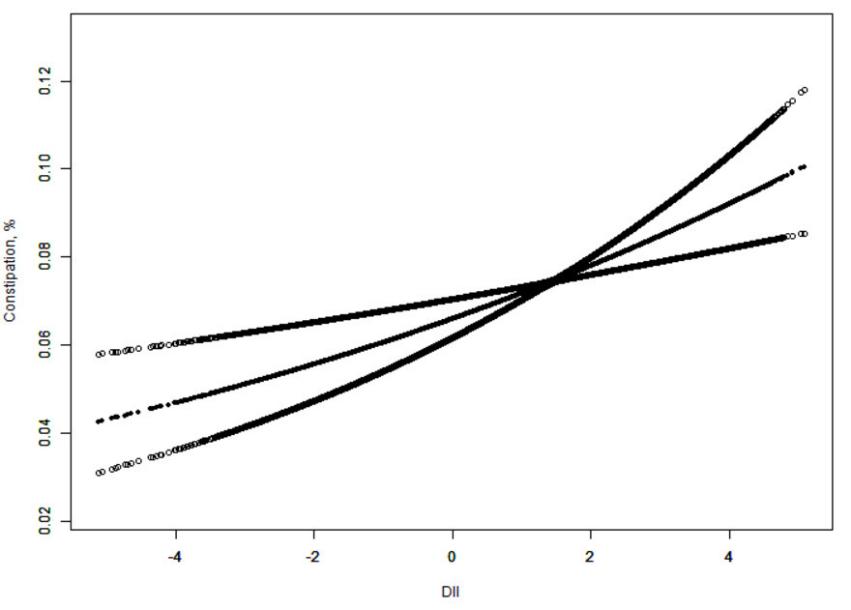


**Supplementary Figure 1** Association between DII and constipation.

**Notes:** A relationship between them was detected after adjusting for age; sex; ethnicity; education; marital,smoking; BMI; income-poverty ratio; physical activity; depression; hypertension, tea, coffee, moisture, tap water, bottled water.. A non-linear relationship was found between DII and constipation in the curve fitting diagram. The solid line in the middle represents the smooth curve fit between variables. Imaginary lines represent the 95% of confidence interval from the fit.

**Abbreviations:** DII, dietary inflammatory index. BMI, body mass index.

**
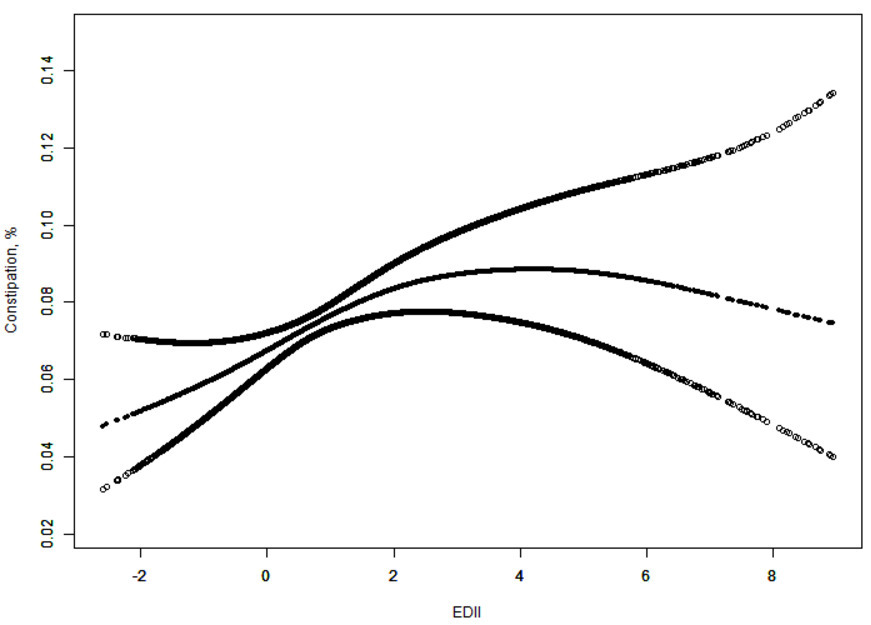
**

**Supplementary Figure 2** Association between E-DII and constipation.

**Notes:** A relationship between them was detected after adjusting for age; sex; ethnicity; education; marital,smoking; BMI; income-poverty ratio; physical activity; depression; hypertension, tea, coffee, moisture, tap water, bottled water.. A non-linear relationship was found between E-DII and constipation in the curve fitting diagram. The solid line in the middle represents the smooth curve fit between variables. Imaginary lines represent the 95% of confidence interval from the fit.

**Abbreviations:**E-DII, Energy-Adjusted Dietary Inflammatory Index. BMI, body mass index.

**Supplementary Table 1** Association of DII with constipation after imputation, weighted.

| Exposure | Model Ⅰ | Model II | Model Ⅲ |
| --- | --- | --- | --- |
| Overall |  |  |  |
| DII | 1.23(1.16,1.30) | 1.13(1.07,1.20) | 1.13(1.06,1.20) |
| Per-SD increase | 1.45(1.31,1.61) | 1.25(1.13,1.39) | 1.24(1.12,1.38) |
| Q1 | Ref. | Ref. | Ref. |
| Q2 | 1.24(0.91,1.69) | 1.09(0.79,1.51) | 1.12(0.81,1.56) |
| Q3 | 1.71(1.31,2.23) | 1.32(1.00,1.75) | 1.32(0.99,1.77) |
| Q4 | 2.59(1.98,3.39) | 1.78(1.32,2.41) | 1.78(1.30,2.45) |
| Men |  |  |  |
| DII | 1.26(1.12,1.42) | 1.20(1.07,1.35) | 1.17(1.04,1.31) |
| Per-SD increase | 1.51(1.22,1.87) | 1.39(1.13,1.71) | 1.32(1.08,1.62) |
| Q1 | Ref. | Ref. | Ref. |
| Q2 | 1.12(0.62,2.03) | 1.00(0.56,1.78) | 1.00(0.56,1.79) |
| Q3 | 1.59(1.04,2.43) | 1.36(0.88,2.10) | 1.32(0.80,2.19) |
| Q4 | 3.08(1.72,5.53) | 2.50(1.42,4.39) | 2.29(1.32,3.99) |
| Female |  |  |  |
| DII | 1.14(1.06,1.22) | 1.10(1.03,1.18) | 1.11(1.03,1.20) |
| Per-SD increase | 1.26(1.12,1.42) | 1.19(1.05,1.35) | 1.21(1.06,1.39) |
| Q1 | Ref. | Ref. | Ref. |
| Q2 | 1.18(0.83,1.67) | 1.12(0.78,1.61) | 1.54(0.84,2.82) |
| Q3 | 1.40(0.96,2.04) | 1.27(0.87,1.86) | 1.32(0.90,1.91) |
| Q4 | 1.80(1.28,2.54) | 1.56(1.08,2.26) | 1.67(1.12,2.49) |

**Notes:** Model I: no covariates were adjusted.

Model II: adjusted for age; sex; ethnicity; education.

Model III: adjusted for age; sex; ethnicity; education; marital,smoking; BMI; income-poverty ratio; physical activity; depression; hypertension, tea, coffee, moisture, tap water, bottled water.

DII quartile ranges: Quartile 1 = -5.11 - 0.25; Quartile 2 = 1.72 - 2.87; Quartile 3= 1.72 - 2.87,Quartile 4= 2.87 - 5.09.

E-DII quartile ranges: Quartile 1 = -2.60 - 0.11; Quartile 2 = 0.11 - 0.86; Quartile 3= 0.86 - 1.78, Quartile 4= 1.78 - 8.95.

**Abbreviations:** NHANES, National Health and Nutrition Examination Survey; DII, dietary inflammatory index. E-DII, Energy-Adjusted Dietary Inflammatory Index. BMI, body mass index.

**Supplementary Table 2** Association of E-DII with constipation after imputation, weighted.

| Exposure | Model Ⅰ | Model II | Model Ⅲ |
| --- | --- | --- | --- |
| Overall |  |  |  |
| E-DII | 1.22(1.16,1.28) | 1.11(1.05,1.17) | 1.09(1.02,1.16) |
| Per-SD increase | 1.34(1.25,1.44) | 1.16(1.07,1.25) | 1.14(1.04,1.25) |
| Q1 | Ref. | Ref. | Ref. |
| Q2 | 1.17(0.84,1.62) | 1.07(0.77,1.48) | 1.08(0.76,1.53) |
| Q3 | 1.87(1.40,2.50) | 1.43(1.06,1.93) | 1.44(1.05,1.97) |
| Q4 | 2.57(1.93,3.41) | 1.69(1.24,2.31) | 1.74(1.22,2.47) |
| Men |  |  |  |
| E-DII | 1.34(1.18,1.53) | 1.27(1.11,1.45) | 1.24(1.09,1.41) |
| Per-SD increase | 1.53(1.28,1.83) | 1.41(1.16,1.71) | 1.36(1.13,1.63) |
| Q1 | Ref. | Ref. | Ref. |
| Q2 | 1.14(0.69,1.89) | 1.03(0.63,1.69) | 1.01(0.62,1.66) |
| Q3 | 2.05(1.28,3.29) | 1.74(1.07,2.84) | 1.77(1.01,3.11) |
| Q4 | 3.01(1.49,6.09) | 1.43(1.21,4.89) | 2.24(1.23,4.06) |
| Female |  |  |  |
| E-DII | 1.10(1.04,1.16) | 1.07(1.01,1.13) | 1.07(0.98,1.16) |
| Per-SD increase | 1.15(1.06,1.24) | 1.10(1.01,1.20) | 1.10(0.98,1.24) |
| Q1 | Ref. | Ref. | Ref. |
| Q2 | 1.13(0.75,1.71) | 1.08(0.71,1.63) | 1.13(0.75,1.70) |
| Q3 | 1.41(0.97,2.05) | 1.27(0.88,1.84) | 1.30(0.92,1.84) |
| Q4 | 1.69(1.19,2.41) | 1.48(1.03,2.13) | 1.60(1.05,2.44) |

**Notes:** Model I: no covariates were adjusted.

Model II: adjusted for age; sex; ethnicity; education.

Model III: adjusted for age; sex; ethnicity; education; marital,smoking; BMI; income-poverty ratio; physical activity; depression; hypertension, tea, coffee, moisture, tap water, bottled water.

DII quartile ranges: Quartile 1 = -5.11 - 0.25; Quartile 2 = 1.72 - 2.87; Quartile 3= 1.72 - 2.87,Quartile 4= 2.87 - 5.09.

E-DII quartile ranges: Quartile 1 = -2.60 - 0.11; Quartile 2 = 0.11 - 0.86; Quartile 3= 0.86 - 1.78, Quartile 4= 1.78 - 8.95.

**Abbreviations:** NHANES, National Health and Nutrition Examination Survey; DII, dietary inflammatory index. E-DII, Energy-Adjusted Dietary Inflammatory Index. BMI, body mass index.

**Supplementary Table 3** Table Normal test

| Varibles | Statistic | P value |
| --- | --- | --- |
| Tea | 0.3275 | <0.001 |
| Coffee | 0.2332 | <0.001 |
| Moisture | 0.0903 | <0.001 |
| Tap water | 0.2390 | <0.001 |
| Bottled water | 0.2688 | <0.001 |
| Plain water | 0.1391 | <0.001 |
| Dietary fiber | 0.0542 | <0.001 |
| Energy | 0.0714 | <0.001 |
| DII | 0.0618 | <0.001 |
| EDII | 0.0635 | <0.001 |

**Supplementary Table 4** Baseline characteristics of DII component.

| Variables | Overall |
| --- | --- |
| Energy intake (kcal/day) | 2027.06 ± 825.50 |
| Protein intake (g/day) | 79.59 ± 34.49 |
| Carbohydrates intake (g/day) | 249.08 ± 105.84 |
| Fibre intake (g/day) | 16.21 ± 8.46 |
| Total fat intake (g/day) | 75.74 ± 37.74 |
| Total saturated fat intake (g/day) | 24.80 ± 13.52 |
| MUFA intake (g/day) | 27.75 ± 14.54 |
| PUFA intake (g/day) | 16.48 ± 9.16 |
| Cholesterol intake (g/day) | 281.82 ± 186.09 |
| Vitamin A intake (mcg/day) | 623.90 ± 549.75 |
| Beta-carotene intake (mcg/day) | 2106.07 ± 2853.32 |
| Thiamin intake (mg/day) | 1.60 ± 0.80 |
| Riboflavin intake (mg/day) | 2.09 ± 1.07 |
| Niacin intake (mg/day) | 24.41 ± 12.00 |
| Vitamin B6 intake (mg/day) | 1.99 ± 1.12 |
| 16Folic acid intake (mcg/day) | 186.69 ± 153.96 |
| Vitamin B12 intake (mcg/day) | 5.31 ± 5.44 |
| 18Vitamin C intake (mg/day) | 87.46 ± 78.20 |
| Vitamin D intake (mcg/day) | 4.65 ± 4.19 |
| Vitamin E intake (mg/day) | 7.15 ± 4.55 |
| Magnesium intake (mg/day) | 285.87 ± 123.41 |
| Ferrum intake (mg/day) | 15.23 ± 7.77 |
| Zinc intake (mg/day) | 11.63 ± 7.30 |
| Selenium intake (mcg/day) | 107.73 ± 49.87 |
| Caffeine intake (mg/day) | 153.08 ± 182.76 |
| Alcohol intake (g/day) | 8.16 ± 19.79 |
| N-6 fatty acids intake (g/day) | 16.16 ± 9.03 |
| N-3 fatty acids intake (g/day) | 0.14 ± 0.29 |
